# Factors affecting local plant knowledge in isolated communities from Patagonian steppe: Metacommunity theory is revealed as a methodological approach

**DOI:** 10.1371/journal.pone.0274481

**Published:** 2022-09-12

**Authors:** Flávia Rosa Santoro, Marina Richeri, Ana Haydée Ladio

**Affiliations:** 1 IMBIV-CONICET, Universidad Nacional de Córdoba, Córdoba, Argentina; 2 Universidad Nacional de la Patagonia San Juan Bosco, Puerto Madryn, Argentina; 3 INIBIOMA-CONICET-Universidad Nacional del Comahue, S.C. Bariloche, Argentina; Universidad Mayor de San Andrés, PLURINATIONAL STATE OF BOLIVIA

## Abstract

The Patagonian steppe is a refuge for several indigenous peoples who live in relatively isolated communities, depending heavily on natural resources for their activities, health, and food security. The local ecological knowledge is a reservoir that generates full wellbeing and for which it must be the object of protection and local development. In this study, we aimed to find which factors can influence local ecological knowledge from a metacommunity on the Patagonian steppe. We analyzed variation in knowledge about cultivated and gathered plants used as medicinal, edible, and firewood according to multiple factors widely discussed in the ethnobiological literature: age, gender, formal education, occupation, indigenous identity, contact with urban centers, use of biomedicine, hunting, and handcrafted textile production. We conducted semi-structured interviews with local experts, accessed by the snowball technique. We found that formal education is a key factor in the variation of local ecological knowledge among people. In addition, we found that knowledge varies between people who practice activities inside and outside the home, concentrating knowledge between cultivated and gathered plants, respectively. Our urbanization proxies did not point to an influence of this factor on local knowledge, but specialists living in a larger community with signs of internal urbanization processes had much less knowledge. Our results allowed us to visualize the importance of studying metacommunities as a whole, to verify complexities and intersections of overlapping factors. Studies in metacommunities open up a range of possibilities for ethnobiological analysis.

## Introduction

The starting premise of this study is based on the rapid and notorious social and environmental changes that affect the entire world, but more significantly, impoverished and isolated communities. In Patagonia, several traditional rural communities, descendants of native peoples, resist and persist in environments considered inhospitable, facing environmental, social, and economic problems, supported mainly by local ecological knowledge about natural resources. Particularly in extra-Andean Patagonia, which includes part of the phytogeographic province of the mountain and steppe, several authors coincide in pointing out that rural communities have suffered successive phenomena of cultural transformation in response to the hegemonic influence of market societies [1–3]. The repeated migratory processes to and from the Patagonian interior [4, 5] have particularly affected the original Tehuelche and Mapuche communities settled in the Patagonian steppe.

In these regions, the rural communities represent “biocultural refuges” [6, 7], places of high retention of social memory and construction of specific knowledge and practices. Such refuges are related to food security, health, and the administration of biodiversity—which are the heritage of a community. These scenarios constitute reservoirs that generate full well-being and for which they must be the object of protection and local development. In this context, plants are not only part of the material elements that people use but also correspond to symbolic, economic, historical, and social aspects [7]. The way men and women of different generations perceive and use plants shape their culture, their ways of life, and the local landscape [7].

Several studies have documented the use of natural resources by the local aged members and warned about the lack of transfer of their knowledge to young members of communities of Patagonian communities [8, 9]. In this sense, some authors suggest that decontextualized formal education—which takes place in rural villages—tends to devalue the local knowledge that children learn informally with their families and encourages the incorporation of concepts that are not their own [10–12].

On the other hand, some authors observed that the degree of contact with the urban lifestyle also influences the local ecological knowledge of plants in Patagonian populations [2, 13]. Another study in the same region shows how knowledge of plants can be different between those people who recognize themselves as descendants of original Mapuche peoples and those who do not [14]. Even the performance of traditional practices, such as hunting, and wild plant gathering, can influence the local ecological knowledge [15]. For Ladio [15], cattle transhumance, hunting, wild plant gathering and plant cultivation form unique, distinct cultural domains that are different from each other and reflect different contexts of knowledge acquisition.

Taking into account the importance of sociocultural factors in the distribution of local ecological knowledge, we aim to analyze the influence of a series of multiple factors on the local ecological knowledge of plants (cultivated and gathered) for medicinal, edible and firewood uses in traditional communities in the Patagonian steppe. Knowing how the local ecological knowledge is organized and distributed in traditional populations allows us to visualize a picture of the dynamics of this knowledge and makes it possible to think about strategies for its maintenance. With our results, we intend to discover which factors can demonstrate a possible loss of knowledge. Furthermore, discovering patterns among different human populations allows us to draw theoretical insights into human behavior.

Our study setting is three traditional communities that together form a metacommunity (according to the conceptual design proposal by Albuquerque et al. [16]) in the Patagonian steppe, in the Comarca de la Meseta Central del Chubut, Argentina. The three communities are relatively isolated from other populations and are so intertwined that many people were born in one community but live or have families in another. From this perspective, we will analyze communities separately, considering that they may have intrinsic properties of metacommunities. Most of its inhabitants are descendants of native peoples of Mapuche and/or Tehuelche origins but people who do not recognize themselves as coming from the original peoples also live and have a strong bond with the region [7]. The communities are relatively far from urban centers, but there are two large cities (approximately 200 and 450 km distant from the communities) that are usually visited by some residents. Like many populations of Argentine Patagonia, these communities have based their survival on multiple strategies with rationality proper to the social economy. Domestic animals are used for self-consumption and wool production for yarns, a traditional practice among the settlers. The hunting of wild animals is also practiced to complement the consumption of proteins and as a form of maintaining a traditional practice [3, 7, 17].

Considering the multifactorial nature of our study, we believe that the background of each of our hypotheses would be easier to visualize if it were systematized in a box. Therefore, in Box 1, we present each of our variables, what the main studies have found about their relationship with local ecological knowledge of plants, and what we hypothesize. We believe that finding relationships between this diversity of factors and traditional ecological knowledge about plants can signal what can be done to mitigate the loss of knowledge caused by any of these factors. Safeguard traditional knowledge is essential for the autonomy of native peoples in the Patagonian steppe, especially in the current context of such rapid and constant sociocultural and environmental changes.

**Box 1. Factors that could affect plant local ecological knowledge in communities from Central Meseta of Chubut (Argentina)**

**Age**—Several studies suggest a positive relationship between the number of known resources and the age of those who know them [9, 18]. Many scientists have assumed that older people, as they have more time interacting with natural resources, have more knowledge than young people (for a discussion on the topic, see Torres-Avilez et al. [19]). In this sense, we hypothesize that age influences local botanical knowledge. Therefore, we expect that we expect that the older the age, the greater the number of known plants in all categories.

**Gender**—Differences in plant knowledge between men and women have been partly explained as a consequence of the division of labor in domestic and productive tasks and also due to historical gender divisions in access to natural resources [20]. However, these differences vary greatly between local populations [21]. Considering these results, we hypothesized that gender, as it represents a division of labor, influences local botanical knowledge. We expect that there is a difference between the genders according to the different categories of plant use (from which we cannot predict the direction), showing a division of labor.

**Formal education**—Bringing together a small literature review and an important case study, Reyes-García et al. [22] show that formal education has a negative effect on local ecological knowledge, with rare exceptions (see, for example, Ruiz-Mallenet al. [23]). Considering these findings, we hypothesize that formal education influences local botanical knowledge. We expect that knowledge of plants in all categories will be negatively affected by the length of formal education.

**Occupation**—Some studies that find a relationship between formal education and knowledge of plants propose that this negative relationship arises because people with different formal education levels have different occupations. People with less formal education tend to have occupations linked to traditional land use, which would make them more knowledgeable about plants, while people with more formal education have non-traditional activities, leading to less knowledge about plants [24, 25]. In our study, we seek to analyze whether these two variables are interconnected, but regardless of this relationship, considering these suggestions above, we hypothesize that occupation influences local botanical knowledge. We expect that people who work with non-traditional activities know fewer plants in general than people who work with traditional activities.

**Indigenous identity**—Based on studies on the difference in knowledge between people of different indigenous identities living in the same place [26, 27], but specifically a previous study in Patagonia that shows a significant difference in medicinal plant knowledge between people of Mapuche and non-Mapuche origin living in the same place under the same conditions [14], we hypothesize that indigenous identity influences local botanical knowledge. We expect that people who declare themselves to be of Mapuche and/or Tehuelche origin will present greater local ecological knowledge about plants in general than people who do not consider themselves to be of native origin.

**Contact with urban centers**—Previous studies on urbanization and knowledge of plants in general, measured through different variables, found different relationships [28–30]. However, as suggested by Ferreira Junior et al. [31], by putting together a discussion on this topic, showing the three possible relationships (positive, negative, or neutral), it seems reasonable to associate the low knowledge of natural resources in a community with the proximity to urban centers, since urbanization provides a series of alternatives to human subsistence that could lead to the abandonment of traditional practices. Considering this competition between the availability of resources, we hypothesize that contact with urban centers influences the local botanical knowledge. We expect that people who visit urban centers more frequently have less knowledge about plants for different uses.

**Use of biomedicine**—In a very similar way to what happens with studies on access to urban centers, studies that deal with the use of biomedicine by traditional populations reach different conclusions, depending on how they assess this relationship ([32, 33]; for ex.). Therefore, following the same logic as the topic above, the idea of competition between different types of resources, we hypothesize that use of biomedicine influences the local botanical knowledge. We expect that people who claim to use pharmaceutical remedies will have less knowledge specifically about medicinal plants.

**Hunting**—There is evidence in the literature of Patagonia that hunting is extremely linked to greater plant knowledge [34], mainly regarding the wild flora [15]. In this sense, and considering that hunting is an activity intrinsic to the cultural traditions of the populations we are studying, we can hypothesize that the practice of hunting influences the local botanical knowledge. We expect that that people who hunt know more about plants in all use categories, especially gathered ones.

**Handcrafted textile production**—Handcrafted textile production -a process that involves raising livestock, spinning the yarn, and the product fabric—represents an important attribute of cultural heritage and identity in peasant and Mapuche communities of Patagonia. In addition, the extensive use of these textiles as clothing due to harsh climate conditions has probably allowed for a thorough exploration of the dyeing plants and an extensive wild plant knowledge [35, 36]. Considering that traditional activities can be related to traditional ecological knowledge, we hypothesize that handcrafted textile production positively influences plant knowledge in general. We expect that people involved in handcrafted textile production know more plants in all use categories.

## Methodology

### Study area and local population

The fieldwork was carried out in three rural communities located in the north-central region of the province of Chubut, Argentina: Lagunita Salada, Gan Gan and Gastre. These small communities are located in plateau landscapes far from the main provincial water courses and are connected to each other by Provincial Route No. 4 (gravel road) and local roads. Access to this area is usually difficult and sometimes interrupted by the presence of snow or the impact of rain during several months of the year, mainly from June to October.

The vegetation of the region corresponds to the complex of the Patagonian steppe. The most conspicuous shrubs have numerous adaptations to strong winds from the west and extreme aridity, such as the “quilimbai” (*Chuquiraga avellanedae* Lorentz), the “matalaguna” (*Lycium ameghinoi* Speg.), the “yaoyín” (*Lycium chilense* Bertero), the “privet” (*Mulguraea ligustrina* Lag. N.O’Leary & P. Peralta) and”carob”(*Prosopis denudans* Benth.) [37, 38]. In the lower areas, it presents mallines: very humid soils with a great development of grasses, located along permanent or semi-permanent water courses, and dead-end basins where water accumulates. The mallines receive both groundwater and surface water and serve as a resource for livestock and the settlement of villages. Steppe vegetation withstands low temperatures, strong winds and low rainfall, and often develops on soils covered by boulders.

People from those communities live in a relatively isolated form from other villages and need to draw lots of difficult environmental and social conditions. The participants of our study are mainly descendants of Mapuche and/or Tehuelche peoples. People who do not recognize themselves as coming from native peoples also participated. These people are descendants of foreigners from other countries and other regions of Argentina, but who have been settled in the region for more than a generation [7].

The Mapuche peoples are considered the largest indigenous nation in southern South America [38]. In *Mapudungun*, the mapuche language, “Mapuche” means people of the land. These populations correspond to different enclaves and origins with a strong dependence on the resources of the Andean temperate forests [38]. The Mapuche have suffered many changes in their customs over time, including forced migrations to regions with less environmental offer, such as the region of our study [4, 39, 40]. The "Tehuelches septentrionales australes" or "Gününa Këna " peoples, also called "Patagones" by the Europeans, originally inhabit the Patagonian steppe region [41]. The name Tehuelche means "brave people" or “people from the sterile land” in *Mapudungun* [41]. These local populations were part of a regional group of hunter-gatherers who occupied and used the Patagonian lands.

If we consider the history of the original peoples of Patagonia, we can say that the genocide of settlers, the territorial usurpation and the inequitable redistribution of the remaining land of low productive value to the original settlers, the introduction of a capitalist model of production based on sheep farming for industrial production, and more recently, the development of new metalliferous extractive activities, among others, have determined forceful changes in the lives of the inhabitants, both cultural and economic [42, 43]. On the other hand, the installation of new inhabitants in the region and, consequently; of a series of institutions (schools, courts, police, health posts and other representatives of the provincial state) have given rise to and have shaped the current structure of the communities that are part of this study.

We want to emphasize that the three communities are united—since pre-Hispanic times—by intense transhumant activities. The exchange activity between ethnic groups and other populations in the Patagonian region was fundamental, and represented the only solution to face the harsh climate and drought [43]. Today, these three communities are quite isolated from other human populations but extremely interconnected with each other. Many of the people are born in one of the three communities but migrate to others. Despite this connection, each maintains particular characteristics. Based on this, we can say that these three communities together form a metacommunity, according to the proposal by Albuquerque et al. [16]. Considering this perspective, we will analyze communities separately, taking into account that they may have intrinsic properties of metacommunities. We will discuss this perspective more throughout this manuscript.

Below, we describe a little of the peculiarities of each of the protagonist communities of our study:

### Lagunita Salada

Its name comes from a depression located to the east of the village that, according to the local elders, a few decades ago contained salt water in large quantities and permanently. The first school that gave rise to the town was founded on the shore of that lagoon. Today, the site of the lagoon only contains water after an abundant and lasting rain has fallen (R. Ñancutil, resident of Lagunita Salada).

Lagunita Salada is the smallest of the three communities, with 36 houses [7]. However, it has a large investment in social assistance by the provincial government since 2003, which generated the construction of two large buildings for community use, the implementation of the “Plan Calor” (“Heat Plan”, for free distribution of firewood), and subsidies for drought and ash zones, among other projects [7]. Firewood is often the main means of heating and cooking in this village. Access to Lagunita Salada in particular is more difficult than the others since much of the route is made up of rocky soil in many places and clay soil in others. Many households are, in turn, more than 40 km of winding road away from the village or the nearest neighbor. Lagunita is located 194 km from Esquel and 294 km from Trelew, which are the closest urban centers [7].

### Gastre

The name derives from the *Gününa këna* language (voice of Tehuelches) Gástrrek, which designates a bush spread in the region (Azorella monantha Clos, "firewood-stone"). The original peoples used its ground root to eat mixed with grease or water [43].

If we take into account the location of the three sites studied, Gastre represents the westernmost commune. It is located 210 km from Esquel and 453 km from Trelew. It is distinguished by presenting a large number of elevations (hills and mountains) in which cattle (sheep or goats) are usually dispersed. Gastre presents a more continuous relief than Lagunita Salada and its roads, although they are made on rocky soils, do not present great difficulty for most vehicles. Thus, although the peripheral homes are very isolated, they can be visited by relatives or by staff of the regional hospital. Gastre comprises 43 houses. The health post, the multipurpose room (for parties and sports) and the school are the last constructions carried out by the provincial government in the 2000s. Gastre is a village with intense sheep slaughter activity, which is why it is a common image to find leather hangers after butchering. Finally, we highlight that the presence of natural springs and mallines in good condition is characteristic of this site.

### Gan Gan

The Gününa-këna (Tehuelches setentrionales australes), original people of the central plateau of Chubut, called with the voice "gan gan" to the pastures (huncos) that usually grew in the mallines and, particularly, those that abounded in the -called today- "Mallín de los Cual” [43]. Gan Gan represents the most populated and commercially active community, with 140 houses. At its entrance is a boulevard where the "Mallín de los Cual" appears as an arid and hostile micro-site, commonly affected by strong winds and overloaded with cattle. This boulevard is sometimes a scene of social protests. It counts with a tree-lined central plaza and a modern hospital from which ambulances and medical services depart for more isolated homes. Formerly, Gan Gan was a place of crossroads, as it was the center of union of several routes that went to both the mountain range (the Andes) and the sea (the Atlantic). It is located 285 km from Esquel and 339 km from Trelew, which are the closest urban centers.

In addition to being the most numerous localities, Gan Gan is the one with the greatest connectivity between institutions, in relation to the central village and its periphery. Hospital doctors, health stalls, the police and the judges visit the entire area with a certain frequency and relative continuity. Gan Gan is also the center of ordinary and annual assemblies of indigenous peoples, calling assembly members from various neighboring places.

### Ethics approval and consent to participate

This study was conducted according to the ethics guidelines of the ISE Code of Ethics and Nagoya Protocol (Argentinian National Law N.°27246). Before the interviews, we explained the general objective of the research and clarified that their identities would be preserved. We continued with the interview only if the informant agreed to participate in the survey. Therefore, all informants orally confirmed free and informed consent prior to data collection. No other specific additional procedure is mandatory for this kind of study in our country. It was not possible to submit the project to the analysis of an ethics committee. There are no ethics committees in Argentina to deal with studies that involve human populations, only with studies that involve animals.

### Data collection

We carried out field campaigns between 2009 and 2014 in which we interviewed 69 rural settlers (23 in each community) selected by the snowball technique [44]. We used semi-structured interviews [44], in which we inquire about the plants used in the daily life of the family, their different uses (for medical, edible, and fuel use, for example), and the environments in which it is possible to obtain them. During the interviews, we also asked about socioeconomic data and cultural activities, such as age; formal education; gender; occupation; indigenous identity (if they recognize themselves as Mapuche and/or Tehuelche); use of biomedicine and industrial drugs; how often they visit urban centers; and whether they participate in textiles production and hunting, traditional activities in the region.

In addition to the interviews, we carry out participatory workshops with the communities [44]. The participatory workshops were part of the process of return and exchange of the research results. But also, in these workshops, we were able to confirm to which botanical taxons the popular names of the plants cited in the interviews corresponded, eliminate synonyms, and gather other information about the perception of the residents about the plants and their knowledge about them. We carried out different activities to apply the techniques mentioned by the interview participants, as part of the process of return. In Gan Gan we made pañil ointment (Buddleja globosa Hope), in Lagunita Salada we made jarilla ointment (Larrea nitida Cav.) and in Gastre we prepared a natural dye with onions (Allium cepa L.). The attendance at each workshop was varied. In Gan Gan we had the participation of almost 50 residents, in Gastre 25 neighbors attended, and in Lagunita Salada 15 members of the community participated. The application of different strategies allowed us to triangulate the registered information and have a greater scientific rigor in determining the species and their uses [44].

### Data analysis

Our study makes a quantitative analysis of the influence of the sociocultural factors on the number of plants cited by the participants. Therefore, we organized the plants mentioned among those used for firewood, food, medicine, and others (with a very low number of citations, such as ornamental, construction, etc.). Furthermore, we separate cultivated and collected plants. Participants were classified according to their age, gender (w-women or m-men), occupation, education, place of residence among the three communities, frequency of visiting urban centers, indigenous identity (original and non-original peoples), use of biomedicine (0 or 1), and the practice of hunting (0 or 1) and handcrafted textile production (0 or 1). The occupation was divided into 2 categories, related to traditional activities—which included housekeeping, cooking, raising goats, weaving, farmyard, and those people who responded that they had no formal occupation -, and non-traditional—which included those who were employed in municipal or outsourced, such as merchants and hospital, commune and school employees. Formal Education was divided into 3 categories that were numerically analyzed (0- no formal education, 1- primary education, 2- secondary education). The frequency with which they visited urban centers was measured according to the participant’s perception of their visits to cities in the last 5 years. It was classified into 4 categories analyzed numerically (0-no visits, 1-few visits, 2- constant visits, 3-frequent visits).

Therefore, our categorical independent variables were gender, occupation, indigenous identity, use of industrial drugs, community of residence, hunting practice, and handcraft textile production. The numeric independent variables were age, formal education, and contact with urban centers. Our response variables were the total amount of plants, the number of cultivated plants, the number of plants collected, and the number of plants in each category of use (specifically firewood, edible and medicinal) mentioned by each participant. We submitted the data to Spearman’s correlation test to assess whether the numeric predictor variables are correlated and to the Kruskal-Wallis test to assess whether the categorical variables are related to each numeric variable. None of the tests showed any relationship between our variables. Therefore, we put all the independent variables in the same Generalized Linear Model (GLM). Accordingly, we built six models: a model whose response variable was the total number of plants per informant, a model only for cultivated plants, a model for collected plants, one for medicinal plants, one for edible plants, and finally, one for firewood plants. We use Poisson distribution, as the data obtained do not follow a Gaussian distribution. We also used stepwise regression to obtain the best model based on the Akaike Information Criterion (AIC). All analyzes were performed using the R version 3.6.1 program [45], with a significance level of 5%.

## Results

### Overview of people and plant knowledge in the studied communities

The group that participated in our study is composed of the majority of women (Table 1), with an average age of about 50 years. 50% of participants are over 50 years old. A considerable proportion of the participants practice hunting (Table 1)—mostly women, who correspond to 70% of people who practice hunting -and handcraft textile—also mostly women, with 75%. Few people attend urban centers regularly. All data on the population profile of each location according to the sociocultural factors studied are in Table 1. The same table also contains the number of plants mentioned in each community. In total, the participants know 117 useful ethnospecies. The medicinal use category has the highest richness (77 spp., 66%), followed by edible (44 spp., 38%), and fuel (28 spp., 24%) uses.

**Table 1:** Number and proportion of people of each community for each sociocultural aspect and average number of cited plants with standard deviation (SD).

**Sociocultural aspects/number and proportion of people**	**Lagunita**	**Gastre**	**Gan gan**	**Total (proportion)**
Age (average in years)	57 (28–91)	44.96 (18–80)	47.3 (19–81)	49.8
Women	16	16	12	44 (63.8%)
Men	7	7	11	25 (36.2%)
No Formal education	8	12	10	30 (43.5%)
Primary education	13	6	11	30 (43.5%)
Secondary education	2	3	2	9 (13%)
Occupation (traditional work)	14	15	12	41 (59%)
Occupation (non-traditional work)	9	8	11	28 (41%)
Mapuche-Tehuelche ancestry	16	16	19	51 (74%)
Non-native ancestry	7	7	4	18 (26%)
People who use biomedicine	17	18	15	50 (72%)
Null contact with cities	5	9	4	18 (26%)
Little contact with cities	13	7	11	31 (45%)
Frequent contact with cities	3	5	7	15(22%)
Constant contact with cities	2	2	1	5 (7.2%)
Hunting	9	9	8	26 (38%)
Handcrafted textile production	8	8	12	28 (40%)
**Average number of cited plants / Standard Deviation**	**Lagunita Mean (SD)**	**Gastre Mean (SD)**	**Gan gan Mean (SD)**	**Total Mean (SD)**
Total plants	20.2 (6.99)	23.6 (14.85)	13.6 (7.68)	19 (11.17)
Gathered plants	15.9 (5.69)	16.2 (6.69)	9.3 (6.06)	13.9 (6.52)
Cultivated plants	4.3 (4.90)	7.4 (10.92)	3.7 (5.00)	5.2 (7.55)
Medicinal plants	8.9 (4.28)	8.5 (4.68)	5.4 (4.05)	7.6 (4.56)
Edible plants	2.3 (1.28)	1.0 (0.97)	2.0 (1.41)	1.8 (1.33)
Firewood plants	3.4 (1.07)	4.6 (2.67)	0.8 (0.42)	2.9 (2.31)

The maintenance of health is the main focus of the usefulness of plants in the communities studied. Most of the people (50, 72%) affirmed that they also use industrial remedies, coming from biomedicine, in addition to plants to treat locally recognized diseases. However, in the workshops, we were able to record that the majority of the participants considered that plants are more effective than drugs prescribed by doctors or bought without a prescription, in city pharmacies or local stores. In addition to their high effectiveness, the low or no cost of medicinal plants was one of the most frequent arguments to explain the preference to “cure with weeds” in these communities.

Regarding edible plants, we observed that the traditional diet of the Patagonian inhabitants is based on the intake of meat and baked goods, and to a lesser extent, on the consumption of vegetables. In the workshops and the field observations, we were able to register that, in the communities, food is associated with sheep, goat, and game meat, but never with vegetables. Instead, plants are often perceived as entities that accompany the lives of the inhabitants, cure their ailments, and allow them to fight against low temperatures by providing warmth and shelter. In Table 1, we can see that the average citation of these plants was very low.

The fuel category is represented mainly by woody species that are collected for heating and/or cooking food. Few species of fuel use are cultivated. It should be noted that growing trees represents a great demand for irrigation water and few families in the community have adequate water availability to sustain these species. In general, in addition to having the firewood collected from the field and the limited supply of cultivated trees, some residents obtain firewood from the “Provincial Heat Plan” that aims to provide fuel to the most destitute homes in the province, and also by buying firewood and charcoal from local stores.

The gathered plants (in general wild native plant species) have greater utilitarian and cultural importance than cultivated ones. At the workshops [7], we were able to verify that the plants "from the field", the plants that coexist with the inhabitants throughout their lives, that do not require special care, are the most respected and loved. It is common for the men and women of the communities to carry twigs of some gathered plant in their saddlebags or bags, in addition to having them hanging on the walls of the house as a symbol of protection and respect. The participants of the workshops also revealed to us that the youngest learn to locate the collection places of native plants when they accompany the older ones on collection walks or help them by preparing the plants for drying and storing them at home. In these areas of direct learning, children gradually recognize the species that their family has been selecting for a long time. In general, few people know cultivated plants (mostly of edible use) and among those who do, they know few plants.

### Results of the tested hypotheses

Among our predictions, the variables that most explained the variation in most models were formal education (negatively) and community of residence (with less knowledge in Gan gan) (Table 2).

**Table 2:** More explanatory generalized linear model between sociocultural variables and knowledge of plants, AIC value, estimated regression parameters, z values and p values^1^.

Dependent variables	Independent variables	AIC	Explanatory Model		
			Estimate	z	p
Total plants	Intercept	638.97	2.5894447	15.197	< 2e-16
	Formal education	638.97	-0.2130694	-3.545	0.000392*
	Contact w/cities	638.97	0.0321824	0.925	0.354735
	Indigenous Id. (non-original)	638.97	-0.0864753	-1.258	0.208557
	Age	638.97	-0.0004177	-0.180	0.856996
	Comm:Gastre	638.97	0.6080918	8.037	9.19e-16*
	Comm:Lagunita	638.97	0.4769037	5.989	2.12e-09*
	Gender (m)	638.97	0.0971224	1.509	0.131333
	Hunting (1)	638.97	-0.1105447	-1.616	0.686981
	Handcrafted textile (1)	638.97	0.0931212	1.512	0.130620
	Occupation (trad)	638.97	0.1960125	2.798	0.005139*
	Biomedicine use (1)	638.97	0.0537360	-0.813	0.416450
Cultivated	Intercept	713.31	0.72937	2.227	0.025954 *
	Formal Education	713.31	-0.22860	1.888	0.059015
	Contact w/cities	713.31	0.03968	0.593	0.553445
	Indigenous Id. (non-original)	713.31	-0.20772	-1.498	0.134094
	Age	713.31	0.00829	1.867	0.061889
	Comm:Gastre	713.31	0.85281	6.004	1.92e-09 *
	Comm:Lagunita	713.31	0.24409	1.551	0.120973
	Gender (m)	713.31	0.33332	2.636	0.008385*
	Hunting (1)	713.31	-0.64485	-4.821	1.43e-06*
	Handcrafted textile (1)	713.31	0.39179	3.267	0.001086*
	Occupation (trad)	713.31	0.45976	3.531	0.000414*
	Biomedicine use (1)	713.31	-0.20115	1.591	0.111676
Gathered	Intercept	472.05	2.467250	12.359	< 2e-16*
	Formal education	472.05	-0.210240	-3.029	0.00246*
	Contact w/cities	472.05	0.019775	0.482	0.62969
	Indigenous Id. (non-original)	472.05	-0.033820	-0.426	0.67045
	Age	472.05	-0.003733	-1.366	0.17200
	Comm:Gastre	472.05	0.511551	5.699	1.21e-08*
	Comm:Lagunita	472.05	0.554991	5.969	2.38e-09*
	Gender (m)	472.05	0.017861	0.237	0.81232
	Hunting (1)	472.05	0.100082	1.239	0.21546
	Handcrafted textile (1)	472.05	-0.008322	-0.115	0.90827
	Occ.(trad)	472.05	0.071958	0.860	0.38971
	Biomedicine use (1)	472.05	0.011418	0.146	0.88383
Medicinal	Intercept	443.42	1.946766	7.239	4.53e-13 *
	Formal education	443.42	-0.252587	-2.662	0.00776 *
	Contact w/cities	443.42	-0.002862	0.052	0.95847
	Indigenous Id. (non-original)	443.42	-0.134745	-1.227	0.21966
	Age	443.42	-0.005461	-1.478	0.13947
	Comm:Gastre	443.42	0.472744	3.891	9.97e-05 *
	Comm:Lagunita	443.42	0.606394	4.891	1.00e-06 *
	Gender (m)	443.42	0.150015	1.493	0.13541
	Hunting (1)	443.42	0.041396	0.377	0.70652
	Handcrafted textile (1)	443.42	0.017289	0.177	0.85925
	Occ.(trad)	443.42	0.112122	1.328	0.18433
	Biomedicine use (1)	443.42	0.004982	0.047	0.96235
Edible	Intercept	233.75	0.754423	1.370	0.1707
	Formal education	233.75	-0.112545	0.599	0.5491
	Contact w/cities	233.75	0.151414	1.225	0.2206
	Indigenous Id. (non-original)	233.75	0.289755	1.385	0.1660
	Age	233.75	-0.003439	-0.458	0.6468
	Comm:Gastre	233.75	-0.798654	-3.048	0.0023*
	Comm:Lagunita	233.75	0.006391	0.029	0.9772
	Gender (m)	233.75	-0.264473	-1.211	0.2257
	Hunting (1)	233.75	0.149867	0.684	0.4938
	Handcrafted textile (1)	233.75	-0.132435	-0.648	0.5170
	Occ.(trad)	233.75	0.038563	0.167	0.8671
	Biomedicine use (1)		0.133190	0.611	0.5413
Firewood	Intercept	233.75	0.161208	0.335	0.7373
	Formal education	233.75	-0.221927	-1.460	0.1443
	Contact w/cities	233.75	-0.032616	-0.357	0.7208
	Indigenous Id. (non-original)	233.75	0.094981	0.545	0.5860
	Age	233.75	-0.003617	-0.586	0.5578
	Comm:Gastre	233.75	1.716959	6.524	6.83e-11*
	Comm:Lagunita	233.75	1.466980	5.265	1.40e-07*
	Gender (m)	233.75	-0.273046	-1.592	0.1114
	Hunting (1)	233.75	0.480281	2.586	0.0097 *
	Handcrafted textile (1)	233.75	-0.019959	-0.125	0.9004
	Occ.(trad)	233.75	-0.325715	-1.655	0.0979
	Biomedicine use (1)		0.062635	0.354	0.7232

^1^The base levels for analysis of each categorical variable were: Indigenous identity- Mapu-Tehuelche origin; Community—Gan gan; Gender—Women; Hunting—non-hunter; Handcrafted textile—non-weaver; Occupation—non-traditional; Biomedicine use—not using biomedicine.

The effects (Estimate) show how much each other level differs from these base levels. For ex., the estimates 0.6080918 for Gastre and 0.4769037 for Lagunita shown on the table indicate how much Gastre and Lagunita differ from Gan gan (positively) in terms of total plant knowledge. The z value is the number of standard deviations from the mean value. A positive z-score indicates the raw score is higher than the mean average. The asterisk shows the results considered significant, with p< 0.05.

Regarding the general knowledge of plants, the most important variables to be considered were formal education, with a negative relationship (z = -3.545, p = 0.000392), and occupation, in which people who have work linked to traditional practices—such as raising goats, weaving, and farmyard—have greater plant knowledge (z = 2.798, p = 0.005139). In addition, Gan Gan participants were shown to have significantly less overall plant knowledge than the other two populations studied (z = 8.292, p < 0.0001 for Gastre; z = 5.978, p <0.0001 for Lagunita).

For knowledge about cultivated plants, the occupation linked to traditional practices (z = 3.531, p = 0.000414), the male gender (z = 2.636, p = 0.008385), and the practice of handcrafted textile production (z = 3.267, p = 0.001086) showed a positive influence. On the other hand, hunting is negatively related to knowledge about cultivated plants (z = -4.821, p <0.0001). Formal Education did not show a significant negative influence but resulted in a p so close to 0.05 (z = 1.888, p = 0.059015), that we believe it is important to emphasize.

The knowledge of gathered plants followed the same pattern as the general knowledge of plants, with a smaller representation of the Gan gan community (z = 5.699, p <0.0001 to Gastre and z = 5.969, p <0.0001 to Lagunita), and a great negative influence of formal education (z = -3.029, p = 0.0024). The same happened with the specific knowledge of plants in the medicinal category (z = -2.662, p = 0.00776 for formal education and z = 3.891, p <0.0001 for Gastre in relation to Gan gan and z = 4.891, p <0.0001 to Lagunita in relation to Gan gan) (see Table 2).

Specifically for plants used for firewood, hunting has been shown to have a positive influence (z = 2.586, p = 0.0097), and again the population of Gan gan has shown to have a much lower knowledge (Table 2). For edible plants, there was no relationship to be highlighted, except the least knowledge by the population of Gastre (see Table 2). It is important to emphasize that the last two categories had little representation of the number of plants (as shown in Table 1), and their result should be interpreted with caution.

## Discussion

### The high influence of formal education on local ecological knowledge

In a general analysis, formal education proved to be the most important predictor of local ecological knowledge about plants in the studied communities, since this variable predicts knowledge of plants in general, collected and medicinal plants (the categories with the highest number of plants). Western-style formal education is argued to display systemic racism, foster separation from traditional knowledge, and/or impact their integrity [23, 46, 47].

It has been argued that the introduction of traditional knowledge in formal contexts of education may increase rates of intergenerational knowledge transmission, empowering local students and maintaining individual and collective cultural identity [48]. For example, in a study in public schools located in an area of traditional populations on the coast of southeastern Brazil, van Luijk et al. [12] show that a considerable part of the local ecological knowledge held by children is taught at school by their teachers.

Ladio and Molares [48] had already emphasized the role of teachers’ knowledge about the local environment for multicultural integration. The authors found that the local ecological knowledge possessed by Patagonian teachers seems to consist of a body of knowledge constructed on a foundation of accumulated experience of the local environment and the cultural values that have prevailed since the initiation of formal education. The authors also found that the teachers’ traditional botanical knowledge was mainly concentrated among cultivated plants and had less knowledge about wild plants. Perhaps this is the reason why formal education has had a lesser (non-significant) negative influence on cultivated plants.

During our study, rural schools in the province of Chubut, fortunately, counted with programs that include learning in Mapudungun (the Mapuche language) as part of the optional curriculum offered to students and their families. At the time, the strategy was very recent and exploratory and possibly was not in force when our participants (all over 18 years old) attended school. Although it is not enough if isolated from other integration practices, the initiative could contribute to the recreation of knowledge and practices that were intentionally canceled together with the local languages. According to Ladio [15], the Mapuche language includes a complex range of denominations involving ecological, symbolic, and utilitarian aspects, constituting an essential for the community, a unique vehicle that cannot be translated into another culture. Therefore, the incorporation of the Mapuche language in formal education can lead to the acknowledgment of traditional knowledge.

Some studies that found similar results to ours considered that formal education is a covariate that acts together with occupation on local ecological knowledge [23, 24]. Our results could be related to the fact the more formal western education people have, the higher they were likely to get in non-traditional occupations. This could be driven by globalization and de-agrarization processes currently taking place in the area exacerbated by growing rural influence on market society [17].

Therefore, it would not be only the formal education that would make people distance themselves from local ecological knowledge, but also the occupation (which requires more time of formal education) related to work outside the traditional context. In our study, even though the occupation variable had no relation to the level of formal education, we saw that people who work with traditional activities have the greatest knowledge about plants in general and cultivated plants, corroborating this idea. To sum up, a trade-off could exist for the time allocated to formal education and non-traditional activities versus the time allocated to the acquisition of plant knowledge. This aspect should also be considered to a larger extent to understand the complexity of this chain of relationships.

We do not measure the income variable, but there may be an influence chain between formal education, occupation, and income (see [24]). Possibly people with non-traditional occupations, because they are merchants or government employees, have a higher income than others. Higher-income can mean greater access to other non-traditional resources that can be purchased and replace traditional resources, such as food on the market, cooking gas in substitution for firewood, and pharmacy medicines in local stores. For ex., we found that the low cost of medicinal plants was used as an argument to explain the preference to “cure with weeds” in these communities.

### Other predictors of local botanical knowledge: The importance of activities inside versus outside home

Moving on to other explanatory variables of local botanical knowledge among categories, we found a significant difference concerning hunting, in which people who practice this activity know less about cultivated plants than those who do not practice it. On the other hand, handcrafted textile production proved to be a predictor of knowledge of cultivated plants. Our result also shows that people who hunt know more about firewood plants, which are in general gathered plants (although there was no relation between hunting and gathered plants in general). In contrast, textile handcraft is a traditional practice that is carried out inside the home, the same as plant cultivation.

Therefore, we attribute these differences found in the knowledge of cultivated versus gathered plants to the practice of activities near and far from the domestic environment. People who go out hunting, in general, are more active outside the home, dealing with wild plants and animals, and therefore have less knowledge about the resources that are cultivated and more knowledge about gathered plants [15, 34]. Similarly, Patagonian textile production is associated with the use of dye plants, many of them cultivated in orchards and domestic surroundings, as is also the case in other parts of the world [49]. These findings show that knowledge about plants depends on the activities and the place where they are culturally developed. These places are specific contexts of learning and appropriation necessary for the maintenance and innovation of plant knowledge.

Our results also showed that the people of Gastre cited more about cultivated plants than the other communities. From field observations in the three communities, we can say that the wild environment of Gastre is the most deteriorated by overgrazing. Therefore, the role of the gardens can be key to people’s knowledge about plants in this community. However, considering that gathered plants have much greater cultural importance than cultivated ones in all communities—according to field observations and in terms of cited plants—the results on the knowledge of cultivated plants must be relativized. Then, we again emphasize the role of schooling and the possible chain of influence that schooling reflects on occupation with traditional and non-traditional activities—which also shows activities inside and outside the home—on local ecological knowledge.

### Variables that did not influence our results: The importance of different proxies

In general, gender and age did not influence people’s plant knowledge, showing the importance of local cultural context. The only exception was cultivated plants, in which men know more plants than women. Studies examining the connections between gender, age, and plant knowledge find mixed trends, showing the difficulties in trying to define general and homogenous patterns across cultures and locations [18–21]. Indeed, an analysis of the literature shows that a pattern becomes clear when examining the gender division of labor, gendered access to a home garden or wild resources, and gendered control over subsistence and income derived from them [21, 50].

In contrast to our results, most of the literature suggests that women know more about cultivated plants than men because they are more involved in domestic environments [19, 51]. This aspect was not analyzed in depth in this work, so we will be cautious not to fall into gender bias. However, it is important to highlight that, in the studied communities, men and women share some important cultural activities, as hunting and textile handcrafts.

Indigenous identity did not also influence any of the categories of use. We could cautiously say that in the Patagonian steppe, these variables do not explain the variation in traditional plant knowledge, as occurs in Andean Patagonia [14]. One factor that usually lies behind the cultural differences in the local ecological knowledge refers to a longer time of living and experience of native ethnic groups in the study places, which allows for a greater accumulation of knowledge [14, 52]. Another is the difference between social groups in contact due to different cosmologies, i.e. distinct intrinsic values as part of the ancestral realm [53]. In this case, the region has a long history of cultural exchanges and impositions with settlers, European immigrants, and outsiders that became locals, an aspect that could explain the results. On the other hand, we cannot fail to mention possible processes of cultural homogenization in plant knowledge among these groups and communities, as occurs in other indigenous communities around the world [54].

Another expected effect that was not observed in our study is the urbanization effect. Different variables are used as proxies to evaluate the urbanization effect on local ecological knowledge in ethnobiological studies, including the proximity to urban centers [28, 55], the frequency with which inhabitants frequent urban centers [7], and access to so-called "Western" resources, such as the very use of biomedicine [30]. In this study, we evaluated the use of biomedicine and the frequency with which people visit urban centers, since the distance to them was very similar between the three communities, and the frequency of visits reflects the real access that people have to cities. Contrary to what we expected, neither the frequency of visits to large urban centers nor the use of pharmaceutical drugs influenced plant knowledge.

About the concomitant use of medicinal plants with biomedicine tools, the literature brings controversial results [32, 33]. However, the most recent studies show that there can be a great dialogue between the two forms of knowledge, even showing a positive relationship between the two types of knowledge [25, 33]. In our study, we did not find any relationship between knowledge of plants and the use of biomedicine, but we must emphasize that our interviews only accessed whether or not the participants use biomedical resources (0 or 1), but they did not quantify this use. It is possible that there are different degrees of use and access to biomedicine and these different degrees are related to a different knowledge of medicinal plants.

Likewise, the frequency of visits to large urban centers, such as the cities of Trelew and Esquel, proved to be irrelevant to knowledge about plants. At this point, we believe that there is another effect masking urbanization: each community’s urbanization process [55]. Access to large cities is not affecting local knowledge, but probably one of the communities (Gan gan) is undergoing an internal urbanization process that is affecting local ecological knowledge. The fact that Gan gan is larger than the others, with a greater amount of resources besides the traditional ones (since commerce is much larger in this community) and greater connectivity between public institutions (hospital doctors, health stalls, frequent visits from the police and the judges) shows us that this community is facing socioeconomic and cultural changes related to an urbanization process.

In addition to having less knowledge of plants in general, the inhabitants of Gan gan also have less knowledge in gathered, firewood, and medicinal categories, which may be an indication that mainly in these categories there are other types of resources intended for the same use, such as the use of accessible cooking gas and a variety of biomedical drugs. Considering the intense commercial activity of Gan gan (compared to the other communities), we can say that purchasable resources may be competing with collected natural resources since they are more easily obtained. Over time, people tend not to prioritize information in their memory that is not used daily, and this is reflected in less knowledge about natural resources.

From the initial premise of our study, we can say that the lesser knowledge of plants shown by the inhabitants of Gan gan can pose a threat to their livelihoods. This threat is compounded when we realize that the population of Gan gan is increasingly dependent on resources that come from an external rather than a local economy, and less and less dependent on local resources. Considering the current economic crisis in Latin American countries, in which Argentina stands out [56], depending on purchasable resources places the inhabitants in a situation of great vulnerability.

In this sense, Ladio [15] has already demonstrated quantitatively how populations living in arid Patagonia are at less risk of losing their capacity for self-sufficiency if they do not abandon their traditional ways of using the environment. Thus, we highlight the urgency of decision-making bodies to create strategies to maintain the knowledge of traditional communities of the Patagonian steppe—the integration of local ecological knowledge with formal education could be one of them.

### Proposals for future studies: Metacommunities in ethnobiology

The result that we had not foreseen—which was analyzed only because we were working with three different communities—is the large difference in local ecological knowledge between Gan Gan and the other locations. As we discussed, the possible explanation for this result is the urbanization degree of this population in relation to others, and the consequent access to other types of non-traditional resources. Other processes may be behind the lesser knowledge of Gan gan specialists, such as the effect of population size, which can facilitate or hinder different biases of cultural transmission [57].

This quantitative difference in knowledge among communities drew our attention to the dynamics of a metacommunity. Our analysis of these communities from the perspective of metacommunity was inspired by the work of Albuquerque et al. [58], in which the authors bring different scales of ethnobiological studies, from local to global, passing through metacommunities, which can be defined as “a set of distinct human groups living in a region where they can interact with each other” [58]. Therefore, a metacommunity could be treated as a unit of analysis. It is important to say that the proposal by Albuquerque et al. [16] does not refer to the ecological metacommunities model proposed by Leidbold et al. [58], which deal with interactions of populations of different species, but approaches the idea of metapopulations [59].

According to Albuquerque et al. [16], the study of metacommunities can bring new perspectives on the dynamics of local ecological knowledge, which cannot be captured in local studies. We could see differences concerning different degrees of urbanization and competition for resources that would not be observed if we analyzed only one of the communities. However, this discussion on the perspective of metacommunities can be deepened. Thus, we bring some of the ecological concepts of metapopulations to broaden this debate and propose the use of metacommunities in future ethnobiological studies.

The theory of metapopulations was proposed by Levins [59], shortly after the theory of island biogeography [60], to overcome the greatest deficiency of the classical models of population dynamics. A metapopulation is therefore a "population of populations"—a group of local populations connected by migrations [59]. The theory of metapopulations gained importance for conservation when studies in areas of small dimensions found that such areas were the only ones that contained populations (also small) of certain species, being, therefore, the most suitable for their conservation [61]. This finding changed the previous perspective based on the theory of island biogeography, which considered that the larger the fragment, the better for conservation [62]. Therefore, it has alerted conservationists to the need to preserve small fragments [62]. Indeed, the simplest metapopulation models seek to predict the permanence of species in a region. Using simple mathematical models, it is possible to verify that the chances of persistence of a population are much higher if it occupies more than one fragment, regardless of the size of these fragments [60].

As well as species habitats have suffered several fragmentation processes as a result of environmental problems, highlighting the relevance of metapopulation studies in ecology, rural and indigenous communities have also suffered several fragmentation processes resulting from the colonial historical past and present de-agrarization processes in the region. Thus, communities that were once large are now small, and the interconnection of these small communities with others with the same characteristics, forming a metacommunity, can be essential for the maintenance of local ecological knowledge.

Metacommunities in ethnobiology could be seen as communities that have similar cultural characteristics, separated from each other, but with a large flow of people and/or information. Based on ecological metapopulations models, we can say that the chances of persistence of a cultural tradition—as local ecological knowledge, practices, and beliefs—are higher if it is shared by more than one community, regardless of the size of these communities. Furthermore, the migration of information between communities from a metacommunity is essential for the maintenance of this tradition.

Eventually, very small communities from a metacommunity have unique and rich local knowledge that deserves to be preserved. If we only looked at the community of Gan gan, for example, we could be dealing with a much less significant richness of botanical knowledge. Thus, we call attention to future ethnobiological studies focusing efforts on seeking communities adjacent to a given target community, which may represent, together with the first chosen target one, a metacommunity. The study in metacommunity can bring more reliable results in the observation of a phenomenon and show an overview of how knowledge is organized between different related communities.

Following the analogy with the ecological field, the metapopulation models have very rich theoretical and mathematical contributions that can be adapted for use in ethnobiology. Therefore, we want to encourage that this perspective can be taken by ethnobiologists, to obtain new data that can predict the conservation of local ecological knowledge.

## Limitations

Our major limitations are methodological, as we have already pointed out in the discussion. The main one is to use the number of plants known per person. Although a proxy variable widely used in ethnobotany, this method leaves out practices, values, and associated knowledge that are invisibilized, partitioning people’s plant knowledge from their integrity. Additionally, we recognize the limitation of the use of the proxy variables: contact with cities, and biomedicine use, since they might not necessarily capture the complexity, for example in the case of the real use of biomedicine in association with medicinal plants, or about the simplistic way to consider contact with western society as an adverse context in any case. This limits our discussion as we have little precision about the relationship found between these variables. In addition, we work with local experts, and our discussion is limited to this sector of the population, not extending to all inhabitants, who may behave differently. Despite these limitations, we believe that our data on three communities that together form a metacommunity are of great contribution to understanding the conservation status of local plant knowledge in the region.

## Conclusions

The present study gives an overview of the main factors affecting plant knowledge among members of Meseta of Chubut. The main predictor of local botanical knowledge variation is the level of formal education. The integration of formal education with traditional plant knowledge and practices could improve and preserve the local ecological knowledge. We also emphasize the importance of carrying out cultural activities close to or far away from home and in the wild environments. We point out that analysis from the perspective of metacommunities can reveal important regional processes that could not be seen if we analyzed only one community. Furthermore, we call attention to the importance of smaller communities, with less access to purchasable resources, for the maintenance of local ecological knowledge of the entire complex of metacommunities. Finally, we suggest that ethnobiological studies adopt the perspective of metacommunities, to obtain more robust data that can predict the conservation state of local ecological knowledge.

## Supporting information

S1 File Original data analysis worksheet. The name of the participants was protected, and numbers were assigned to each informant. Gender: m-man; w-woman; Identity: mapu- Mapuche origin, teh- Tehuelche origin, mapu-teh—Mapuche and Tehuelche origin, no orig—Non-native ancestry; Use of industrial drugs (biomedicine): 0- people who do not use industrial remedies, 1- people who use industrial remedies; hunt: 0- people who do not hunt, 1- people who hunt; Handcrafted textile: 0—people who do not practice Handcrafted textile; 1—people who practice Handcrafted textile; visits to large cities: null- Null contact with cities, little-little contact with cities, frequent- frequent contact with cities, constant—Constant contact with cities; all—number of all useful plants (number of firewood plants+ edible plants medicinal plants + other uses plants).
(XLSX)

S1 Data (DOCX)
