# Supplementary material for: Factors affecting local plant knowledge in isolated communities from Patagonian steppe: Metacommunity theory is revealed as a methodological approach
Source: PLoS One. 2022 Sep 12;17(9):e0274481. doi: 10.1371/journal.pone.0274481 (PMC9467323; doi:10.1371/journal.pone.0274481)
Supplement: S1 Data — (DOCX) [file pone.0274481.s002.docx]

Inclusivity in global research

PLOS’ policy on inclusivity in global research aims to improve transparency in the reporting of research performed outside of researchers’ own country or community and ensures that PLOS publications reporting global research adhere to high standards for research ethics and authorship. Authors of relevant research articles may be asked to complete the questionnaire below, which outlines ethical, cultural, and scientific considerations specific to inclusivity in global research. This questionnaire may be requested when researchers have travelled to a different country to conduct research, if research uses samples collected in another country, research with Indigenous populations or their lands, or if research is on cultural artefacts. Researchers travelling to another country solely to use laboratory equipment will not normally be required to complete the questionnaire. However, the questionnaire can be requested at the journal’s discretion for any submission – if you have been requested to complete this questionnaire by the PLOS journal you submitted to, please do so.

Please complete the questionnaire below and include this as a Supporting Information file with your manuscript. Note that if your paper is accepted for publication, this checklist will be published with your article in the supporting information files. Please ensure that you reference the checklist in the main body of your manuscript. We suggest adding a subsection ‘Inclusivity in global research’ to your Methods section and adding the following sentence: “Additional information regarding the ethical, cultural, and scientific considerations specific to inclusivity in global research is included in the Supporting Information (SX Checklist)”

The questions have been designed to be applicable to a wide range of study types, and there are subsections for both human subjects research and non-human subjects research. If any of the questions are not relevant to your research please mark them as “N/A” as appropriate.

**Ethical considerations, permits and authorship**

*This section is applicable to all research types.*

Provide details as to who granted permissions and/or consent for the study to take place in the Methods section of your manuscript. This should include the names of **all** ethics boards, governmental organizations, community leaders or other bodies that provided approval for the study. If individuals provided approval refer to these people by their role or title but do not list their name(s).

Reported on page number: 8

If there were any deviations from the study protocol after approval was obtained please provide details of these changes in the Methods section of your manuscript.
Did this study involve local collaborators that are residents of the country where the research was conducted or members of the community studied? If you do not have any authors from said communities, please provide an explanation for this below.

Reported on page number: not applicable

This study had local participants who agreed to be interviewed on a voluntary basis, but they were not part of the authorship of the article. At the beginning of the work, it was collectively decided that the results of the research would be returned to the community through workshops on medicinal plants conducted by Marina Richeri.

Everyone listed as an author should meet PLOS’ criteria for authorship and all individuals who meet these criteria should be included in the author byline, rather than the acknowledgements. Authorship criteria is based on the International Committee of Medical Journal Editors (ICMJE) Uniform Requirements for Manuscripts Submitted to Biomedical Journals - for further information please see here: <https://journals.plos.org/plosone/s/authorship>.

**Human subjects research (e.g. health research, medical research, cross-cultural psychology)**

Did you obtain written informed consent from a representative of the local community or region before the research took place? How did you establish who speaks for the community? Details of written informed consent obtained from study participants should be reported separately in the Methods section of your manuscript.

Prior written informed consent was obtained only from some collaborators, especially from Lagunita Salada. The rest of the participants are illiterate and oral consent was obtained with them. The fieldwork was carried out considering each household unit individually, and at the time of the fieldwork there were no local referents. Our work was based on a respectful dialogue of knowledge that has allowed us to continue working in the area with the inhabitants in other research and local development projects.

Translated with www.DeepL.com/Translator (free version)

How did members of the local community provide input on the aims of the research investigation, its methodology, and its anticipated outcome(s)?

The members of the participating communities showed interest in our research and participated in a committed manner in obtaining information. They have not participated in other instances of the work. In addition, several of their members participated in the workshops for feedback and validation of results, generally held in local schools.

When engaging with the local community, how did you ensure that the informed consent documents and other materials could be understood by local stakeholders?

Yes. The objectives of this research were detailed in the written consent form and also transmitted orally to each of the participants. Subsequent workshops have shown that people have been able to appreciate and understand the objectives of this work.

Will the findings of the research be made available in an understandable format to stakeholders in the community where the study was conducted (e.g. via a presentation, summary report, copies of publications, etc.)? Please provide details of how this will be achieved.

Yes. In addition to the workshops mentioned above, copies of the final written work in Spanish were left in the schools, as well as copies of herbaria of the plants collected with their scientific names.

**Non-human subjects research using specimens/ animals collected as part of the study, or those housed in archival collections. Examples include archaeology, paleontology, botany and zoology.**

Did the permission you obtained from a local authority to perform the study include an agreement on access to outputs and benefit sharing? This may include procedures to enable fair distribution of the benefits and resources arising from the research performed. Please include any details of Prior Informed Consent and Benefit Sharing Agreements obtained. These may be required by field-specific regulations, for example the Convention on Biological Diversity (CBD) and the associated Nagoya Protocol.

At the time of the research there were no local authorities, consent was obtained on an individual basis following the Argentine legislation which is a signatory of the Nagoya protocol as explained on page 8.

If the material used in your study was imported, please A) provide the year it was imported and B) indicate whether permits were obtained to import/export the materials used, C) provide details of any permits obtained. If this information is not available, please indicate this.

Not applicable

If you used archival specimens, please state how the material used in your study was acquired by the institute it is held in and provide details of any permits obtained for the original excavations/ sample collection. If this information is not available, please indicate this.

Plant specimens were collected with the consent of the people in each of their properties. This material was deposited in the Botanical Garden of the San Juan Bosco University in Madryn city and herbarium copies were made for the communities.

How was the potential cultural significance of the materials collected in your study to local communities considered in your research design? Were Indigenous peoples and/or local researchers and institutions involved with archaeological excavations / collection of specimens? If so, please provide a description of their involvement.

The cultural significance of plants for people is important because they are part of their daily life, many of the plants are grown in their gardens and others are part of their pasture lands. They were collected following their indications and care. Many of the plants were collected by the participants themselves. Local rules were respected throughout the procedure.

If your manuscript includes photographs of human remains please indicate whether authors obtained permission from descendants or affiliated cultural communities to do so.

Not applicable
